# Stable Single α-Helices Are Constant Force Springs in Proteins*[Fn FN2]

**DOI:** 10.1074/jbc.M114.585679

**Published:** 2014-08-13

**Authors:** Marcin Wolny, Matthew Batchelor, Peter J. Knight, Emanuele Paci, Lorna Dougan, Michelle Peckham

**Affiliations:** From the Astbury Centre for Structural Molecular Biology, University of Leeds, Leeds LS2 9JT, United Kingdom

**Keywords:** Atomic Force Microscopy (AFM), Circular Dichroism (CD), Cytoskeleton, Molecular Dynamics, Structural Biology, Single α-Helices

## Abstract

Single α-helix (SAH) domains are rich in charged residues (Arg, Lys, and Glu) and stable in solution over a wide range of pH and salt concentrations. They are found in many different proteins where they bridge two functional domains. To test the idea that their high stability might enable these proteins to resist unfolding along their length, the properties and unfolding behavior of the predicted SAH domain from myosin-10 were characterized. The expressed and purified SAH domain was highly helical, melted non-cooperatively, and was monomeric as shown by circular dichroism and mass spectrometry as expected for a SAH domain. Single molecule force spectroscopy experiments showed that the SAH domain unfolded at very low forces (<30 pN) without a characteristic unfolding peak. Molecular dynamics simulations showed that the SAH domain unfolds progressively as the length is increased and refolds progressively as the length is reduced. This enables the SAH domain to act as a constant force spring in the mechanically dynamic environment of the cell.

## Teaser

**Background:** Single α-helix (SAH) domains bridge two functional domains in proteins. Their force response is poorly understood.

**Results:** Modeling and experiments show that SAH domains unfold non-cooperatively at low forces and maintain an approximately constant force as they unfold.

**Conclusion:** SAH domains act as constant force springs.

**Significance:** SAH domains are important mechanical elements in proteins.

## Introduction

Stable single α-helix (SAH)^3^ domains are found in proteins from organisms ranging from bacteria to humans and are present in ∼100 proteins in the human genome (1–3). They are unusual in that they have a relatively high thermal stability and are stable over a wide range of salt and pH conditions without neighboring interactions. They are characterized by a high number of arginine, lysine, and glutamic acid (Arg, Lys, and Glu) residues, and their stability is predicted to arise from the (*i*, *i* ± 4) and (*i*, *i* ± 3) intrahelical interactions between either Arg and Glu or Lys and Glu through the formation of stabilizing salt bridges (3). The unusual stability of these domains contrasts with that of most α-helices, which are stabilized by interactions with neighboring secondary structure elements (*e.g.* in globular proteins, bundles, and coiled coils).

Despite their abundance, the functions and properties of SAH domains remain unclear. SAH domains appear to be fairly rigid and resistant to bending as demonstrated by a combination of optical trap, small angle x-ray scattering measurements, and modeling (4). This suggests that they could function as rigid spacers between protein domains as SAH domains commonly bridge two functional domains (1–3). However, it is unclear how the sequence and structure of SAH domains are linked to their physical, dynamic, and mechanical properties. Gaining an understanding of the properties of SAH domains is thus key to elucidating their cellular roles.

The biological function of SAH domains is likely to be determined by how rigid and inextensible they are over their length. Therefore, it is important to determine how well the SAH domains maintain their highly helical structure over a long length of sequence. For example, do they act as a stiff spacer or as a weak extensible element that unfolds at low forces before other regions of the protein unfold? To answer these questions, we characterized a predicted 97-residue-long SAH domain from myosin-10 using a combination of approaches. We determined the secondary structure of the peptide and its thermal melting characteristics by circular dichroism and confirmed that it was monomeric by mass spectrometry. We used single molecule atomic force spectroscopy to determine its unfolding properties, and we used molecular simulation to understand the physical properties that underlie its unique structural features. These results provide novel insight into the properties of the SAH domain and suggest new ideas about its biological role.

## EXPERIMENTAL PROCEDURES

### 

#### 

##### Constructs for Circular Dichroism Spectroscopy

PCR was used to amplify the sequence for the SAH domain used in these experiments using bovine myosin-10 cDNA as a template (a kind gift from Richard Cheney; UniProt accession number P79114; residues 813–909). The SAH domain was cloned into the pET28a SUMO (small ubiquitin-like modifier) vector to introduce an N-terminal His tag and small ubiquitin-like modifier protein for increased expression and solubility. A tryptophan residue was added to the C terminus to enable *A*_280_ concentration measurements.

##### Constructs for AFM Experiments

A modified pET3 vector encoding a pentamer of titin I27 domains (His tag-I27-I27-I27-I27-I27-Cys-Cys) was a kind gift from Dr. David Brockwell, University of Leeds (5). To generate the I27_5_SAH_1_ construct, the SAH domain was cloned between the second and third I27 domains. To generate the I27_5_SAH_2_ construct, an additional SAH domain was cloned into I27_5_SAH_1_ between the first and second I27 domains.

##### Protein Expression

All proteins were expressed in *Escherichia coli* BL21 Rosetta 2 (Novagen) and purified using nickel-nitrilotriacetic acid affinity and ion-exchange chromatography. For SUMO constructs, SUMO was removed using ULP1 protease before ion-exchange chromatography. The purest fractions were combined and concentrated, resulting in 0.4–1.2 mg/ml protein solutions. Purified protein was dialyzed against 100 mm NaCl, 10 mm sodium phosphate, pH 7.4 and snap frozen in liquid nitrogen for long term storage at −80 °C.

##### Mass Spectrometry

Protein samples (∼0.5 ml; 15–20 μm) were dialyzed (G-Biosciences dialyzers, 1-kDa-molecular weight cutoff) overnight against 50 mm ammonium acetate, pH 7.4 and analyzed by TOF MS analysis (The University of Leeds Mass Spectrometry Facility).

##### Circular Dichroism (CD) Spectroscopy

CD measurements were performed on an Applied Photophysics Chirascan CD spectropolarimeter with a 0.1-cm-path length quartz cuvette in CD buffer (150 mm NaCl, 20 mm Tris, pH 7.4 for pentameric I27 (I27_5_) constructs or 100 mm NaCl, 10 mm sodium phosphate, pH 7.4 for SAH domain). Data were collected every 1 nm with 30-s averaging time with each measurement being an average of two repeated scans. Data presented are averaged from at least three separate measurements of different protein preparations. Thermal measurements were performed in a temperature range from 10 to 85 °C with 0.7 °C/min heating rate with data acquisition every 1 °C and a 20-s averaging time. The sample cooling rate prior to measurement of refolded protein was ∼ 2 °C/min.

To obtain spectra for SAH domains present in the pentameric titin I27 construct, measurements of I27_5_, I27_5_SAH_1_, and I27_5_SAH_2_ at the same molar concentration were performed. The spectrum (millidegrees) of the “empty” I27_5_ construct was subtracted from those of I27_5_SAH_1_ and I27_5_SAH_2_ constructs. The mean residue molar ellipticity of proteins was calculated as described (6). The helical content of proteins was calculated from values of the amide *n*π* transition at 222 nm ([θ_222_]) as described previously (7).

Protein concentration was measured by absorption at 280 nm. Absorption coefficients were obtained from ProtParam software. Concentrations were in the range 1–20 μm.

##### AFM Force Spectroscopy

Protein constructs at ∼0.1 mg/ml in Tris buffer (150 mm NaCl, 20 mm Tris, pH 7.5) were incubated on freshly cleaved template-stripped gold for ∼10 min. Experiments were performed either directly in this solution or after replacement with Tris buffer.

All AFM work was carried out on an Asylum Research MFP-3D instrument operated at room temperature. Cantilevers (MLCT, Bruker) were calibrated prior to collection of force-extension traces using the thermal noise method (8); spring constant values (*k*_c_) were typically 35–45 pN/nm. Force-extension traces were collected at a velocity of 1000 nm/s over a distance of 500–1000 nm with a sampling rate of 5 kHz and a maximum loading force of ∼1.5–3 nN. The position of the tip on the substrate was manually changed every few hundred traces.

To ensure that we only analyzed force-extension traces from a *single protein molecule*, which had been picked up by its N terminus, only traces where five I27 domains were seen to unfold followed by a detachment peak were used. Traces with multiple detachment peaks or where the contour length up to the detachment peak was unphysical (>40 nm more than the fully unfolded length of the construct to allow for errors in origin placement) were excluded. Furthermore, only those traces showing a reasonable fit to a wormlike chain (WLC) model were used. The WLC model links force (*F*) to extension (*x*) and is commonly used to describe the elasticity of single polypeptide chains (9, 10). It has two variable parameters, *p*, the persistence length (a measure of stiffness) and *l_c_*, the contour length (the length the polymer approaches at high force). Using an initial global fit to the five I27 unfolding events, only those traces showing an increase of contour length (*l_c_*) on unfolding titin I27 domains of 28 ± 1 nm and a persistence length (*p*) of 0.4 ± 0.1 nm (11) were used. Model-free values of peak force (unfolding force (*F_u_*)) and the peak-to-peak distance (P2P) were recorded. WLC parameters for a fit up to 100 pN on the first I27 unfolding peak were also recorded. The fit was limited to 100 pN to avoid the onset of the “hump” feature due to I27 unfolding intermediates (12).

The total unfolded lengths of the three different constructs prior to unfolding of the first I27 domain (Table 1, predicted *l*_c_) were calculated from the expected length of unfolded SAH domains and any linker sequences between I27 and SAH domains present in each construct, using 0.38 nm/residue for an unfolded peptide residue (13, 14), together with the length of the folded I27 domains (4.5 nm).

##### Molecular Dynamics (MD) Simulation

The implicit solvent model FACTS (15) was used in conjunction with the united atom CHARMM19 force field for simulations. All simulations were performed at 300 K with Langevin dynamics and a friction coefficient of 3 ps^−1^ (16). The N and C termini of the SAH domain and Ala_97_ and Gly_97_ sequences were capped with acetyl and methylamine groups, respectively (17). The termini of the β-sheet-rich titin I27 domain, taken from the Protein Data Bank structure 1TIT, were uncapped.

An implicit solvent approach was used to characterize the mechanical properties of SAH domains over longer periods of time; their size would make explicit solvation unviable. Although other implicit solvation approaches based on the generalized Born approximation (*e.g.* solvent-accessible surface area and EEF1) (16) showed that SAH domains form highly stable helices, only with FACTS did we observe reversible helix formation for short SAH-like sequences (10–12 residues long).

Starting structures for zero force simulations were prepared by performing a steepest descent minimization (1000 steps) from artificially generated perfectly α-helical conformers of myosin-10 SAH domain, Ala_97_, and Gly_97_ or from the Protein Data Bank structure in the case of I27 followed by a short (100-ps) dynamics run. Perfectly α-helical conformers were created by setting internal dihedral angles to ϕ = −47° and φ = −57°. Zero force simulations lasted at least 100 ns.

Constant velocity force spectroscopy experiments were mimicked in MD simulations by applying an external force between the N-terminal nitrogen atom and C-terminal carbonyl carbon atom as the “cantilever” and moving this away at constant velocity. The value for the single molecule force constant for the springs (30 pN/nm) is realistic and comparable with that of the cantilever used in single molecule force spectroscopy experiments. A range of velocities was used (from 10 to 0.01 nm/ns). These are several orders of magnitude higher than those used in the real experiment (1000 or 10^−6^ nm/ns) due to computational time constraints, and this results in higher values for the unfolding forces *in silico* compared with experiments.

Initial starting structures were prepared by running a short dynamics run at zero force for at least 15 ns except for Ala_97_ for which a 100-ps run was used to enable it to keep its helical structure before applying force. The collapse of the Gly_97_ peptide into a compact random coil allowed us to test the effect of force on the unfolding of a random coil. To remove starting structure bias, a range of starting structures and restart parameters were then prepared by running a sequence of zero force simulations, each lasting 100 ps. Simulations were stopped once a change in the end-to-end distance (distance between the N-terminal nitrogen atom and C-terminal carbonyl carbon atom (*r*_NC_)) of at least 8 nm had been reached. Wordom (version 21) was used to analyze the simulation trajectories (18, 19). Helicity values were calculated using Wordom “dsspcont” assignments of residue secondary structure.

## RESULTS

### 

#### 

##### Residues 813–909 from Myosin-10 Form a SAH Domain in Vitro

**FIGURE 1.**
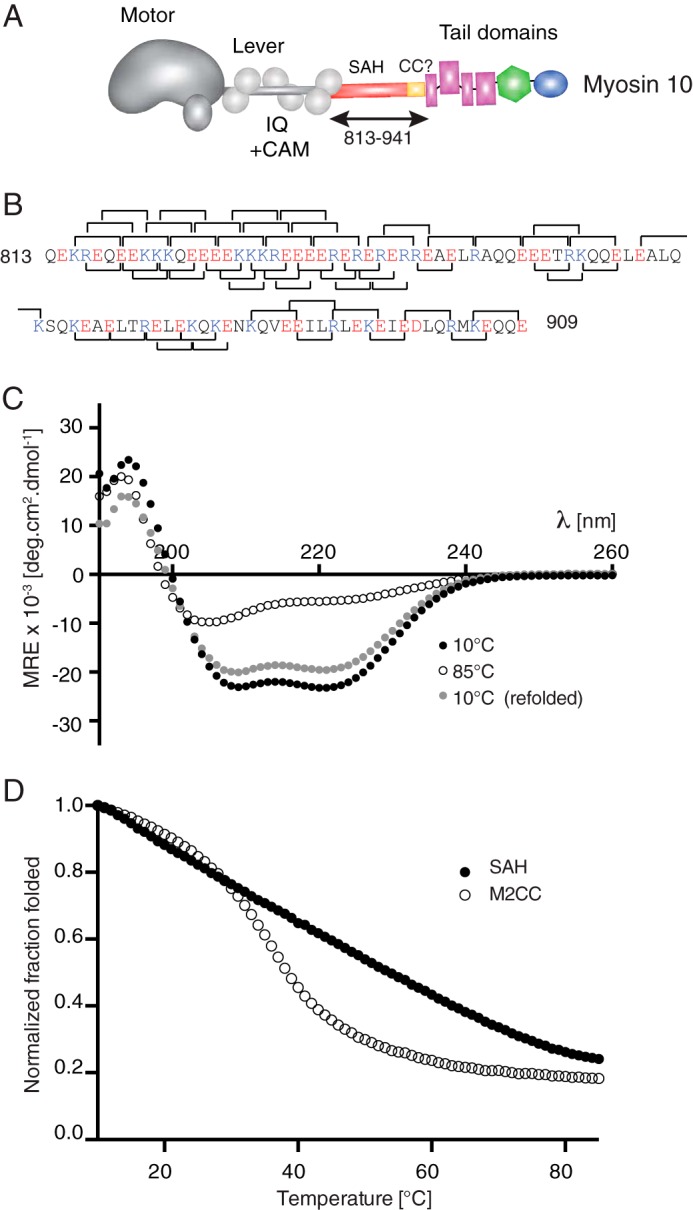
**Intrahelical ionic interactions and circular dichroism of the predicted SAH domain from myosin-10.** *A*, the domains found in myosin-10 showing the position of the predicted SAH domain and the antiparallel coiled coil (*CC*) (residues 813–941) between the lever and the tail domains. *B*, the sequence of the predicted SAH domain used in all the experiments here showing the potential *i*, *i* + 4 interactions and *i*, *i* + 3 interactions as *brackets* above and below the sequence, respectively. Basic residues (Lys and Arg) are shown in *blue*, and acidic residues (Glu and Asp) are shown in *red. C*, mean spectra for the SAH domain from myosin-10 at temperatures of 10 and 85 °C and then after returning to 10 °C. Those collected at 10 °C show profiles typical for α-helical proteins with >80% reversibility after thermal melting. Mean values from *n* = 6 repeats are shown. The helical content was calculated from the measured MRE values at 222 nm to be 67%. *D*, thermal melting curves for the SAH domain compared with that for a known coiled coil (M2CC). For each species, MRE_222_ values have been normalized to the value at 10 °C. *CAM*, calmodulin; *deg*, degrees.

The aim of the experiments reported here was to test the force-extension properties of a SAH domain. We chose to use the putative SAH domain from myosin-10 that is inserted between the myosin head (motor domain and three calmodulin-binding IQ motifs) and the tail domain (Fig. 1*A*). This sequence (residues 808–934; bovine myosin-10) was originally predicted to form coiled coil. However, we showed that a peptide containing the first 36 residues (808–843) is not coiled coil but behaves as a SAH domain (20). The first 6 residues are likely not part of the SAH domain but interact with the calmodulin light chain as part of the final IQ motif (20). In that study, we predicted that the SAH domain was likely to extend beyond the first 36 residues out to residue 908 from our observations of an apparent SAH domain structure by rotary shadowed EM (∼15 nm long; equivalent to ∼100 residues of α-helix). An analysis of this sequence (Fig. 1*B*) shows that although there are fewer charged interactions downstream of the first 36 residues charge-charge interactions are still common, suggesting that this entire region may act as a SAH domain. The distal part of this sequence (residues 883–934) was recently shown to form an antiparallel coiled coil in an isolated peptide, but residues downstream of residue 909 were required for its formation (21). Therefore, we chose to test a peptide from myosin-10 that contained residues 813–909 to confirm that this peptide would behave as a SAH domain (as predicted from our earlier work) before subsequently using this peptide in the force-extension studies.

Mass spectrometry and UV CD experiments showed that as predicted the 97-residue myosin-10 peptide did behave as a SAH domain *in vitro*. The CD spectrum at 10 °C showed the characteristic strong double minima at 208 and 222 nm typical of an α-helix (Fig. 1*C*). The peptide was estimated to be ∼67% helical from the value obtained at 222 nm; this is similar to the helical content measured for the first 36 residues of this SAH domain in earlier experiments (20). Heating the peptide to 85 °C reduced the helical content in a non-cooperative manner (Fig. 1*D*), and this effect was reversed on cooling to 10 °C at which the helical content returned to at least 80% of its original value before thermal denaturation (Fig. 1*C*), consistent with the behavior of SAH domains (20). In contrast, a similar length of coiled coil peptide that contains 15 heptad repeats from β-cardiac myosin 2 (M2CC) showed the typical cooperative melting of a coiled coil (22). Mass spectrometry analysis showed that at a concentration of 15–20 μm the myosin-10 SAH domain was monomeric with a mass of 12.5 kDa, the molecular mass predicted for the monomer, whereas the coiled coil peptide (M2CC) was dimeric with a mass of 25.7 kDa (compared with the predicted molecular mass of 12.9 kDa for the monomer).

##### The SAH Domain Retains Its Helical Properties and Aids Construct Refolding When Inserted between Titin I27 Domains

**FIGURE 2.**
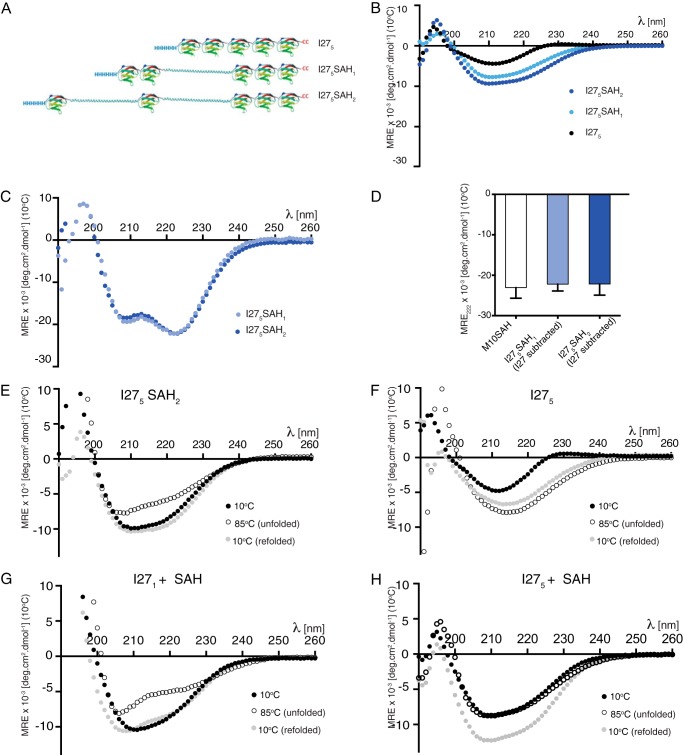
**I27_5_-SAH constructs and their circular dichroism spectra.** *A* shows the three constructs used in these experiments: I27_5_, I27_5_SAH_1_ (which contains a single myosin-10 SAH domain (residues 813–909)), and I27_5_SAH_2_ (which contains two myosin-10 SAH domains inserted between adjacent I27 domains). *B*, mean CD spectra of all three constructs (*n* = 3) at 10 °C. *C*, subtraction of the I27_5_ spectrum from the I27_5_SAH_1_ and I27_5_SAH_2_ spectra reveals the typical spectrum expected for an α-helical structure for both constructs. *D*, a comparison of the MRE values at 222 nm for the myosin-10 (*M10*) SAH peptide and for the SAH domain in I27_5_SAH_1_ and I27_5_SAH_2_. *Error bars* represent S.D. *E* and *F*, spectra for thermal melting experiments using the I27_5_SAH_2_ construct (*E*) and the I27_5_ construct (*F*). *G* and *H*, CD spectra before, during, and after thermal melting for a mixture of monomeric I27 (*I27_1_*) and the SAH domain in a 5:2 molar ratio (*G*) and for a mixture of I27_5_ and the SAH domain (1:2 molar ratio) (*H*). *deg*, degrees.

To perform single molecule force spectroscopy experiments, the SAH domain was inserted between titin I27 domains. The I27 domain was used as a reference protein in these experiments as its unfolding profile in single molecule force spectroscopy experiments has been well characterized (12). Two constructs in which either one or two SAH domains from myosin-10 (residues 813–909) were inserted between titin I27 domains were generated (I27_5_SAH_1_ and I27_5_SAH_2_, respectively; Fig. 2*A*).

Before carrying out the single molecule force spectroscopy experiments, we first used CD to confirm that the SAH domain remained helical when inserted between I27 domains in the I27_5_SAH_1_ and I27_5_SAH_2_ constructs. Their CD spectra are complex as there are contributions from both I27 domains, which have a β-sandwich structure (23), and SAH domains, which are expected to be helical. The spectra show how introducing first one then two SAH domains sequentially into the I27_5_ construct (Fig. 2*B*) decreases the mean residue ellipticity value at 222 nm as expected for the insertion of an α-helix into the I27 construct. Thus, both I27_5_SAH_1_ and I27_5_SAH_2_ contain a mixture of β-sheet and α-helix as expected if the SAH domain remains helical. Subtraction of the I27_5_ spectrum from those for I27_5_SAH_1_ and I27_5_SAH_2_ revealed typical α-helical spectra with two minima present at 222 and 208 nm and a maximum at 195 nm (Fig. 2*C*). The magnitude of these minima is indistinguishable from that observed for the isolated SAH domain from myosin-10 (Fig. 2*D*).

We also found that I27_5_SAH_2_ was able to fully refold after thermal melting (Fig. 2*E*), whereas I27_5_ was not (Fig. 2*F*). In a mixture of monomeric I27 domains and SAH domains in a ratio of 5:2 (the same ratio as in the I27_5_SAH_2_ construct), both peptides were able to refold after thermal melting as the CD traces superpose (Fig. 2*G*). In contrast, a mixture of I27_5_ and SAH domains in a ratio of 1:2 (as in the I27_5_SAH_2_ construct) showed that I27_5_ does not refold after melting. Note that melting of I27_5_ decreases the MRE_222__nm_ value (becomes more negative), whereas melting of the SAH domain increases it. Hence, in this case, the folded (10 °C) and unfolded (85 °C) traces superpose. On refolding, the 222 nm value decreases due to the refolding (decrease in MRE_222__nm_) of the SAH domain only. The inability of I27_5_ to refold likely results from subdomain swapping between the concatenated I27 domains that interferes with the refolding of each individual domain (24–26). However, when SAH domains are inserted between I27 domains, their high thermal stability allows them to keep the I27_5_ domains separate in the I27_5_SAH_2_ construct, enabling them to refold. Taken together, these results suggest that insertion of a SAH domain between two functional domains in any protein would be able to effectively separate the two domains even when they unfold and that this behavior can additionally promote the refolding of these two domains.

##### Single Molecule Force Spectroscopy Shows That the SAH Domain Unfolds Below 30 pN

As the I27_5_ constructs contain five I27 domains, we limited our analysis to those traces that contain five unfolding peaks, one for each of the five I27 domains in the construct, followed by a detachment peak (∼0.03% of the collected traces). This approach ensures that only single molecules, which have been picked up by their ends, are included in our analysis (12). Importantly, this stringent criterion ensures that the unfolding of the SAH domains is included in the traces. Furthermore, a comparison of the traces for constructs containing zero (I27_5_), one (I27_5_SAH_1_), or two (I27_5_SAH_2_) identical SAH domains allows us to be sure that we have correctly identified the contribution of the SAH domain to the trace.

**FIGURE 3.**
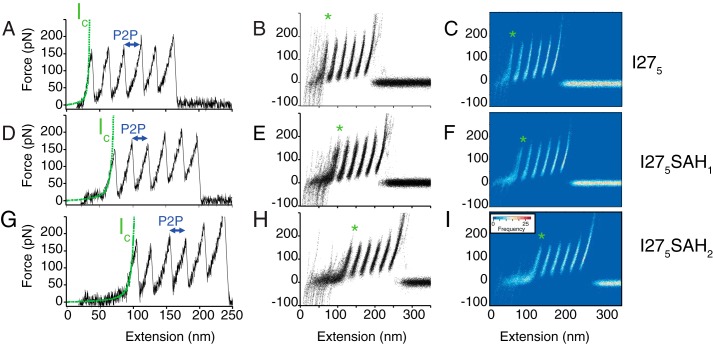
**Single molecule force spectroscopy results for I27_5_, I27_5_SAH_1_, and I27_5_SAH_2_.** *A*, *D*, and *G* show a single example trace for each construct collected at 1000 nm/s for I27_5_ (*A*), I27_5_SAH_1_ (*D*), and I27_5_SAH_2_ (*G*). All three constructs show five I27 unfolding peaks and a clear detachment peak. The *green dashed line* shows the fit of a WLC model to the region of the trace up to 100 pN on the first I27 unfolding peak used to calculate *l_c_*. P2P (*arrow*) indicates the peak-to-peak distance between the I27 unfolding peaks. *B*, *E*, and *H* show an overlay of all the force-extension traces analyzed for I27_5_ (*B*), I27_5_SAH_1_ (*E*), and I27_5_SAH_2_ (*H*). Traces were aligned at 100 pN prior to the detachment peak to apply a consistent approach across all traces and for all constructs avoiding the problem of spurious variations in the early (near zero force) parts of the trace. The *green asterisk* highlights the position of the first I27 unfolding peak. For all three constructs, features observed in the aligned plots are quite variable in the region prior to unfolding of the first I27 domain, but the most densely populated path is via a WLC-like profile. *C*, *F*, and *I* show the equivalent color density plots for each construct (37). Each plot is divided into a grid of 1 nm × 2 pN pixels, and the color indicates the number of points from the force-extension trace overlay that are contained within each pixel. The *green asterisk* highlights the position of the first I27 unfolding peak.

**FIGURE 4.**
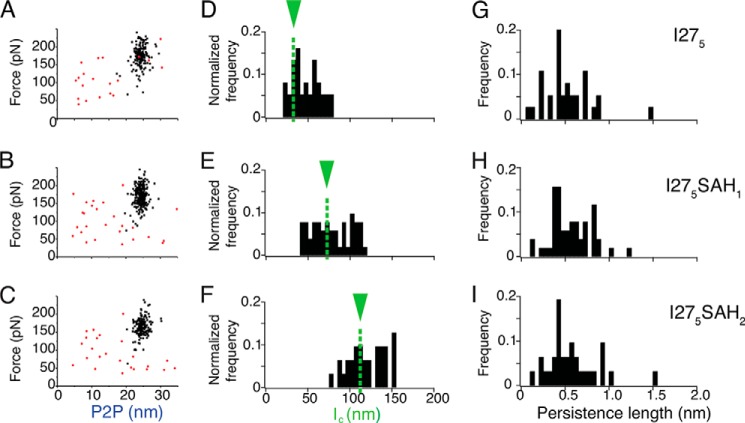
**Analysis of single molecule force spectroscopy results for I27_5_, I27_5_SAH_1_, and I27_5_SAH_2_.** *A–C*, scatter plots of unfolding forces *versus* P2P distances for I27_5_ (*A*), I27_5_SAH_1_ (*B*), and I27_5_SAH_2_ (*C*). The *black squares* show the unfolding forces for the I27 domains, and *red circles* represent any peaks preceding the first I27 unfolding event. The P2P calculated for each construct is shown in Table 1. *D–F*, histograms of *l*_c_ calculated from the WLC fit to the first I27 unfolding event for each of the three constructs. Altering the bin size to 2 or 20 nm yielded similar distributions. The *green arrow* and *green dashed lines* in *D–F*, show the expected contour length values (Table 1), including the number of residues in the SAH domain. *G–I*, associated persistence length histograms describing the wormlike chain fit to the first I27 unfolding event for each construct. The three constructs give very similar distributions for persistence length. Altering the bin size to 0.02 or 0.2 nm gave similar distributions.

The average *l_c_* from a WLC fit up to the first I27 unfolding event in I27_5_ was 47 nm (Fig. 3, *A–C*; Fig. 4*B*; and Table 1). This value is similar to that predicted from the number of amino acids present in linker domains between I27 domains in the I27_5_ construct (Table 1). The measurements of peak-to-peak distance, unfolding force, and persistence lengths calculated from the WLC fit for the I27_5_ construct (Table 1) were all consistent with those reported earlier for I27 domains (12).

**TABLE 1:** **Analysis of traces for I27_5_, I27_5_SAH_1_, and I27_5_SAH_2_ obtained by single molecule force spectroscopy** The experimental results are shown as mean ± S.D. We measured the I27 *F_u_* and P2P, and calculated *l_c_* and *p* from a WLC fit to the first I27 unfolding peak for protein constructs. A pulling speed of 1000 nm/s was used in all experiments. *F_u_* is the maximum force prior to an I27 unfolding event. P2P is defined as the distance from one unfolding peak to the same force value on the following unfolding event; if the following peak is lower than the preceding one then the distance was measured at the height of the second peak. The predicted *l_c_* was calculated from the number of amino acids in the linker and SAH domains, assuming the SAH domains unfold prior to the I27 domains (see “Experimental Procedures”).

Construct	No. traces	*F_u_*	P2P	*l_c_*	*p*	Predicted *l_c_*
		*pN*	*nm*	*nm*	*nm*	*nm*
I27_5_	37	174 ± 29	24.2 ± 1.5	47 ± 16	0.50 ± 0.25	34
I27_5_SAH_1_	51	171 ± 27	24.3 ± 1.2	77 ± 23	0.55 ± 0.22	73
I27_5_SAH_2_	31	165 ± 25	24.6 ± 1.5	120 ± 22	0.57 ± 0.28	111

Importantly, introducing one SAH domain into the I27_5_ construct did not result in an additional unfolding peak but did increase the distance to the first I27 unfolding peak (Fig. 3, *D–F*). The average contour length from a WLC fit to the first I27 unfolding event increased from 47 (I27_5_) to 77 nm (Table 1 and Fig. 4*E*). This increase in contour length is in line with the calculated increase in length that would result from addition of an unfolded SAH domain (Table 1). Similarly, inserting two SAH domains (Fig. 3, *G–I*) increased the distance to the first I27 unfolding peak even further (Fig. 3, *G–I*), increasing the calculated contour length to 120 nm (Table 1 and Fig. 4*F*). These data show that the SAH domains unfold before the first I27 domain unfolds. The lack of an additional unfolding peak is strongly suggestive that the SAH domain unfolds non-cooperatively.

Once the SAH domain has unfolded, the I27 domains unfold as expected for an I27_5_ construct, again supporting our conclusion that the SAH domain unfolds before the first I27 domain. The measured P2P (24 nm; Table 1 and Fig. 4, *A–C*), *F_u_* (∼170 pN at 1000 nm/s; Table 1), and subsequent increase in contour length (∼28 nm) together with the similar values for the persistence length for the first I27 unfolding peak (Fig. 4, *G–I*) for all three constructs were all in good agreement with previous studies (10, 11) of I27 constructs. Taken together, the increase in average contour length before the first I27 peak as first one and then two SAH domains are inserted together with the lack of any additional characteristic unfolding peaks shows that the SAH domain unfolds before the I27 domain unfolds, and it does so over a range of forces below 30 pN (the sensitivity of the instrument).

##### MD Simulations of AFM Experiments Confirm That SAH Domains Unfold Non-cooperatively

To confirm our experimental findings that the SAH domain unfolds non-cooperatively at forces below that at which the I27 domains unfold, we used MD simulations. We compared the dynamic behavior of the myosin-10 SAH domain with that of the monomeric I27 domain (Protein Data Bank code 1TIT) as used in these experiments. In addition, we used the behavior of polyalanine (Ala_97_) as a model of a “normal” α-helix and polyglycine (Gly_97_) as a model random coil. The SAH domain and the Ala_97_ and Gly_97_ peptides were all given α-helical structures at the start of each simulation.

**FIGURE 5.**
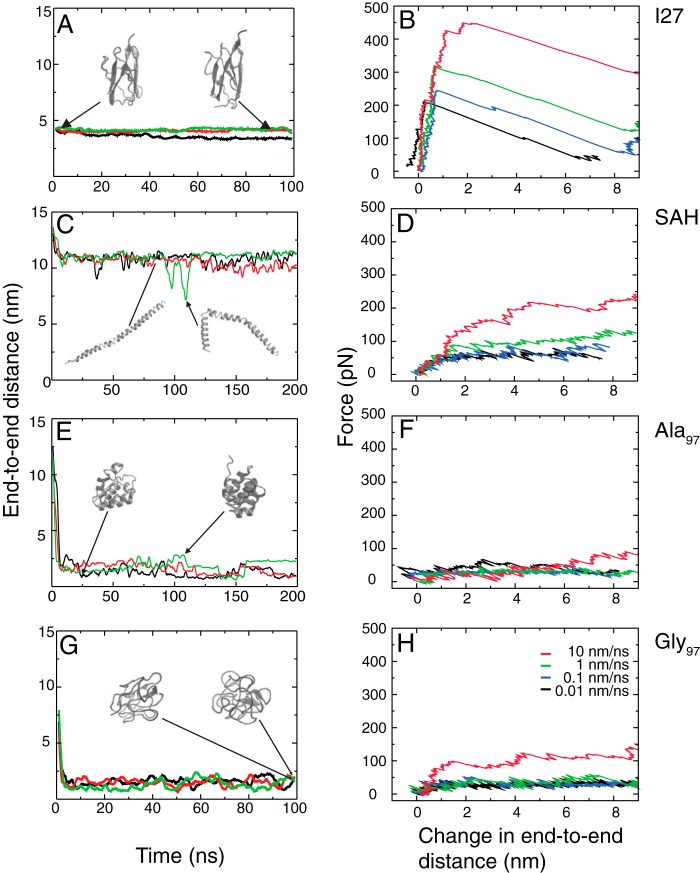
**Simulations of I27, SAH, Ala_97_, and Gly_97_ peptides.** *A*, *C*, *E*, and *G* show the variation in *r*_NC_ as a function of time at zero force. The total length of simulation was 200 ns for the SAH domain and Ala_97_ and 100 ns for Gly_97_ and I27. The data are presented as running averages over 2-ns intervals. Three separate simulations are shown (*red*, *green*, and *black lines*). *Insets* show snapshots of structures from one of the three simulations. The second snapshot in *C* has the lowest *r*_NC_ from all SAH simulations and represents a rarely occurring and transient bent structure. The initial small drop in *r*_NC_ observed for the SAH domain occurs as the peptide initially relaxes from its starting perfect α-helical structure. Example simulations for I27, the SAH domain, Ala_97_, and Gly_97_ are shown as supplemental Movies 1–4, respectively. *B*, *D*, *F*, and *H* show example force-extension traces from MD simulations. Pulling speeds were 10 (*red*), 1 (*green*), 0.1 (*blue*), and 0.01 nm/ns (*black*) and are presented as running averages over 0.01-, 0.1-, 1-, and 10-ns intervals, respectively. The cantilever spring constant (*k_c_*) is 30 pN/nm in each case. Example simulations are shown in supplemental Movies 5–8. The starting structure for I27 is its native structure, and the direction of pull is determined by the vector between the N and C termini. The starting structure for the SAH domain and Ala_97_ is a straight helix, whereas that for Gly_97_ is a compact random coil. As the proteins have different initial lengths and in particular the helices are longer than the folded I27 and collapsed Gly_97_ structures, the data are presented as the change in the end-to-end distance (*r*_NC_) from the initial protein conformer.

The MD simulations showed that the titin I27 domain maintained a structure close to its initial crystal structure throughout the lifetime of the simulation in the absence of any applied force or length constraint as expected (Fig. 5*A* and supplemental Movie 1). In single molecule force-extension simulations, a saw-toothed curve was observed (Fig. 5*B*), consistent with experimental observations. A small change in length as force increased was followed by a large change in length associated with a collapse in force as the protein unfolds (Fig. 5*B* and supplemental Movie 2).

Simulations of the SAH domain in the absence of force showed that it maintained a long helical structure throughout the lifetime (200 ns) of the simulations (Fig. 5*C* and supplemental Movie 3) with an average helicity of 74 ± 6% in good agreement with our experimental finding. The stability of the SAH domain is evidenced by the limited change in end-to-end distance (*r*_NC_) as a function of time. In single molecule force-extension simulations, the initial rise in force was lower than that observed for I27 and was not followed by a force collapse as the peptide lengthened further (Fig. 5*D* and supplemental Movie 4). Instead, the force level remained approximately constant, whereas length continuously increased. This difference was consistently observed over a range of pulling speeds.

Most of the loss in helicity occurred from the ends of the helix during elongation, although occasional unfolding within the helix was also observed. Closer inspection of the simulations showed that it is the break in the hydrogen bond along the helix backbone that is the determining factor prior to extension of the molecule. This break commonly occurs at the same time that the salt bridge between Lys and Glu (or Arg and Glu) pairs is disrupted, although the salt bridges are fairly mobile and can break and reform without a break in the hydrogen bond.

In contrast to the SAH domain, the polyalanine helix (Ala_97_) quickly lost its helical structure (within 7 ns) to form a helical bundle, the precise structure of which differed for each simulation (Fig. 5*E* and supplemental Movie 5). The absence of specific charge-charge interactions between residues as found in the SAH domain means that a single Ala_97_ helix does not remain stable over long periods of time. In the single molecule force-extension simulations, there was very little increase in force as the length increased (Fig. 5*F* and supplemental Movie 6). Gly_97_ rapidly lost its helical starting structure to form a collapsed random coil structure under zero force conditions (Fig. 5*G* and supplemental Movie 7), which is expected because glycines are not usually part of helical structures and favor coil formation. In force-extension simulations starting from collapsed random coil structures, Gly_97_ unfolded non-cooperatively at low forces (Fig. 5*H* and supplemental Movie 8) as expected for a random coil (14).

The behavior of the I27 domain in our modeling nicely matches that observed experimentally in single molecule force spectroscopy experiments. Moreover, the modeling of the SAH domain under zero force conditions shows that this peptide remains helical as observed experimentally by CD, thereby providing validation for the simulation method. At each velocity used, the force required to unfold I27 was consistently greater than the force required to unfold the SAH domain (Fig. 5, *B* and *D*), consistent with our experimental findings. For example, the maximum force values measured at a velocity of 1 nm/ns were 2.3-fold higher for I27 than for the SAH domain. In addition, the forces required to unfold the SAH were higher than those required to extend Ala_97_ and Gly_97_ (Fig. 5, *F* and *H*), demonstrating that the unfolding of the SAH domain is different from that for a random coil (Gly_97_) or an uncharged helix (Ala_97_) and that it shows a higher resistance to extension.

These simulations also demonstrate that the unfolding of the SAH domain is non-cooperative in nature in that after the initial elongation phase the force remained approximately constant as the peptide continued to be extended. This type of behavior is reminiscent of that predicted for a phase transition (27), which occurs when a crystalline structure becomes amorphous when extended and in this equilibrium maintains a constant force while lengthening.

##### MD Simulations of AFM Experiments Show That SAH Domains Refold Progressively

**FIGURE 6.**
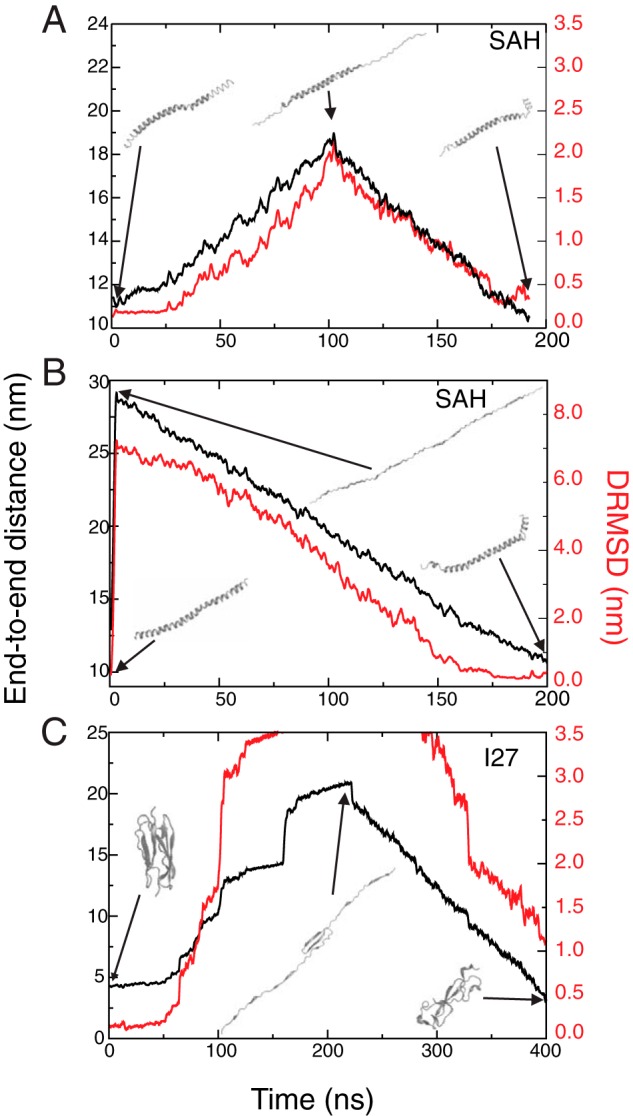
**Simulations that investigate the refolding of the SAH domain (*A* and *B*) and I27 (*C*).** A force was applied to the ends of the peptides by adding springs to the N and C termini and moving them apart at 0.1 nm/ns (except in *B* where it was 10 nm/ns), and this procedure was then reversed so that the N and C termini approach one another at a velocity of 0.1 nm/ns to determine whether the peptides could refold. The force constant for the springs was 30 pN/nm. Plotted on each graph are the end-to-end distances (*black*; *left axis*), which show how the distance between the termini of all molecules first increases during pulling and then decreases on pulling reversal. The DRMSD values, which provide a measure of the similarity of the structure during the simulation to the initial structure, are shown in *red* (*right axis*). *DRMSD* is the root mean square deviation of the internal distances matrix involving the peptide backbone atoms from that computed for the initial structure. Snapshot images of the peptide structures during the simulation (positions indicated by the *arrows*) are shown.

Given the good agreement between simulations and experiments, next the MD simulations were used to predict whether the SAH domain could refold after extension either to a partial helix or a completely extended coil structure as the sensitivity of our single molecule force-extension experiments prevents us from measuring this experimentally for the SAH domain. The simulations showed that the SAH domain was able to refold (Fig. 6*A*) with a distance root mean square deviation <0.5 nm on returning to its initial end-to-end distance after unfolding to a partial helix (length, ∼19 nm) (see example simulation in supplemental Movie 9). Similarly, it was able to refold after rapid extension at 10 nm/ns (Fig. 6*B*) to a completely non-helical chain (∼30 nm) (see example simulation in supplemental Movie 10). In contrast, under the same conditions, I27 does not refold correctly on the same time scale (Fig. 6*C*). Of note is that for I27 the steps observed represent unfolding events (Fig. 6*D*).

## DISCUSSION

These data show that the 97-residue peptide from myosin-10 that we predicted to be a SAH domain based on our earlier work (20) behaves as expected for an SAH domain *in vitro*. It maintains its helical structure when inserted between I27 domains, is effective at separating individual I27 domains, and promotes their refolding after thermal melting. The single molecule force spectroscopy experiments demonstrate that the SAH domain completely unfolds at forces of less than 30 pN at the pulling speed used, and the lack of a characteristic unfolding peak strongly suggests that the peptide is unfolding non-cooperatively. Simulations confirm that the SAH domain unfolds at lower forces than those required to unfold the I27 domain, that it unfolds non-cooperatively, and that uniquely it maintains an approximately constant force during extension. Moreover, the simulations predict that the SAH domain can refold after extension when allowed to shorten back to its original length. These data suggest that the SAH domain does not simply function as a “spacer” separating two functional domains in proteins but has a more complex, force-dependent role.

The ability of the long α-helix of the SAH domain (27 turns) to maintain an approximately constant force while it is extended suggests that the SAH domain behaves as a constant force spring in which the energy stored is linearly dependent on extension. Importantly, this is different from a Hookean spring in which the force rises linearly with extension, and thus the energy stored has a quadratic dependence on extension. If the SAH domain behaved like a Hookean spring as it is extended the resulting restoring force could quickly rise high enough to detach the SAH-containing protein from its binding partner (*e.g.* myosin from its binding site on actin). By functioning as a constant force spring instead, the SAH domain allows a variable separation between the domains it connects without increased risk to the integrity of such macromolecular complexes. Although the distribution of charged side chains along the SAH domain is not uniform, the simulations show a near constant force as the SAH domain is extended. The reason for this is that the force measurements in the simulations are a derivative of the free energy. This free energy contains entropy and is not simply a function of the number of salt bridges (although these do contribute to the total energy). Thus, the near constant force we measure in the simulations is derived from the direct estimation of the probability of all microstates compatible with a given extension.

Interestingly, the force-length behavior of the SAH domain is somewhat similar to that reported for a coiled coil in single molecule force spectroscopy experiments (28). At a force of about 20 pN, the coiled coil was found to extend to about 2.5 times its original length, whereas the force only rose by about another 5 pN. During this force plateau, the coiled coil was shown to completely unfold as the measured change in length was equivalent to the difference in length between a completely helical peptide and a completely extended one. The coiled coil was described as a “truly elastic” protein in that it is able to refold while experiencing forces of up to 30 pN without any detectable hysteresis. Both the coiled coil and the SAH domain are unlike typical elastic materials in being non-Hookean; their elasticity is derived from the helix-extended coil rapid equilibrium.

It is possible that the SAH domain may function in a similar way to the much shorter six-turn α-helical linker between the My12 and My13 immunoglobulin (Ig) domains in the muscle protein myomesin. This helical linker also rapidly and reversibly unfolds at low forces of ∼30 pN (29), and this property is suggested to enable it to act as a stress absorber, protecting adjacent domains from unfolding. It is not clear how much of the unfolding force for the myomesin linker is solely due to helix unfolding as only two of the turns are free of interactions with other parts of the protein (30). Thus, it is remarkable that the much longer SAH construct of 27 turns that we have investigated here is able to show this type of behavior, which is likely to be important for the cellular roles of proteins containing SAH domains.

SAH domains are characterized by a high content of charged residues. Our observation that the long predicted SAH region from myosin-10 forms a SAH domain as does the short 37-residue peptide we investigated earlier (20) indicates that there is no evidence for length dependence for the stability of SAH domains. The lower helical content of the longer SAH peptide from myosin-10 (60–65% helical) compared with the short proximal region (75–80% helical) may stem from a lower overall fraction of charged amino acid residues in the distal region of this longer sequence (65%) compared with the proximal region (83%) that may slightly reduce helix stability. As our construct was missing residues 910–934, which are reported to be required for antiparallel coiled coil formation (21), it is not surprising that our peptide was monomeric. As we know that SAH domains retain their SAH-like properties when placed next to coiled coil domains (31), it is likely that even if this distal part dimerizes in intact myosin-10 as reported the SAH domain extends up to the start of the antiparallel coiled coil.

The unfolding behavior and stability of the SAH domain may arise from the frequent charge-charge interactions, which may provide some resilience to stretching, and from the likelihood that salt bridges break individually. When a turn of helix breaks under the effect of thermal fluctuations in SAH domains, it can reform rapidly even if the local structure is considerably deformed. This explains why SAH domains do not show force peaks in AFM force-extension traces in that any of the salt bridges can break in any order. This is in contrast to the unfolding force profile of the I27 domain in which specific hydrogen bonds between the A′ and G β-strands are thought to break first before the rest of the peptide can be extended, giving rise to the characteristic saw tooth unfolding profile (32).

*In vivo*, in the context of the full-length molecules containing SAH domains inserted between functional domains, this force-length behavior is likely to be important for the cellular role of the protein. How does the SAH contribute to the function of proteins? SAH domains are found in three myosin classes, 6, 7, and 10, which have functions in actin-rich regions of the cell (33, 34), such as the filopodium for myosin-10 (35). To effectively traffic its cargo through a dense actin meshwork *in vivo*, the motor must remain attached to actin even when forces on the cargo/tail domain may rise high enough to potentially detach the motor. Intriguingly, the stall force for myosin-10 is only ∼1 pN (36). We suggest that on the millisecond time scale the SAH domain may unfold even at this low force and continue to unfold such that the force remains low and constant and the motor can remain attached to its track. When forces on the cargo/tail domain are subsequently reduced, the SAH domain would then refold. By extension to the range of other proteins in which SAH domains are found, this could be a general mechanism to ensure that protein domains remain bound to their binding partners even in situations where they experience higher force levels.

Taken together, these and previous data suggest that SAH domains are relatively stable over a broad range of pH and salt concentrations, are relatively rigid under bending stresses (with a bending stiffness of ∼10-fold less than that of the canonical myosin lever (31)), and can maintain a constant force level when stretched. Moreover, SAH domains show a strong propensity to refold and form a long helix when the stress is released. The reversible unfolding and the ability of the SAH domain to maintain force while it is stretched are likely to be important for the biological function of all SAH domains.

## Supplementary Material

Supplemental Data

## Footnotes

3 The abbreviations used are:

SAH: single α-helix
AFM: atomic force microscopy
WLC: wormlike chain
*p*: persistence length
*l_c_*: contour length
P2P: peak-to-peak distance
*F_u_*: unfolding force
MD: molecular dynamics
FACTS: fast analytical continuum treatment of solvation
M2CC: myosin 2 coiled coil peptide
I27_5_: pentameric I27
MRE: mean residue ellipticity
*r*_NC_: distance between the N-terminal nitrogen atom and C-terminal carbon atom.
