# Supplementary material for: Stable Single α-Helices Are Constant Force Springs in Proteins
Source: J Biol Chem. 2014 Aug 13;289(40):27825–35. doi: 10.1074/jbc.M114.585679 (PMC4183817; doi:10.1074/jbc.M114.585679)
Supplement: Supplemental Data [file supp_289_40_27825__index.html]

Stable single alpha helices are constant-force springs in proteins — Stable Single α-Helices Are Constant Force Springs in Proteins — Single α-Helices — Supplemental Data 

# Stable Single α-Helices Are Constant Force Springs in Proteins

## Supplemental Data

**Files in this Data Supplement:**

- Movie Captions (.docx, 78 KB) - Captions for supplemental movies
- Movie 1 (.mov, 2.6 MB) - Movie 1: i27\_no\_force Zero-force simulation of I27 from the PDB structure 1TIT (100 ns simulation time)
- Movie 2 (.mov, 3.9 MB) - Movie 2: i27\_stretch Stretching I27 from a near-native starting structure at 108 nm/s (120 ns simulation time)
- Movie 3 (.mov, 1.6 MB) - Movie 3: SAH\_no\_force Zero-force simulation of M10 SAH from a long helix starting structure (100 ns simulation time)
- Movie 4 (.mov, 1.3 MB) - Movie 4: myo10\_SAH Stretching M10 from a long helix starting structure at 108 nm/s (~100 ns simulation time)
- Movie 5 (.mov, 2.9 MB) - Movie 5: a97\_no\_force Zero-force simulation of A97 from a long helix starting structure (100 ns simulation time)
- Movie 6 (.mov, 1.3 MB) - Movie 6: a97\_stretch Stretching A97 from a long helix starting structure at 108 nm/s (~90 ns simulation time)
- Movie 7 (.mov, 3.3 MB) - Movie 7: g97\_no\_force Zero-force simulation of G97 from a long helix starting structure (100 ns simulation time)
- Movie 8 (.mov, 2.1 MB) - Movie 8: g97\_stretch Stretching G97 from a collapsed coil starting structure at 108 nm/s (100 ns simulation time)
- Movie 9 (.mov, 2.4 MB) - Movie 9: myo10\_stretch\_relax Stretching M10 from a long helix starting structure at 108 nm/s and then reversing the direction of pull (&#x26;ndash;108 nm/s) allowing the protein to refold (~200 ns simulation time)
- Movie 10 (.mov, 2.8 MB) - Movie 10: myo10\_full\_stretch\_relax Stretching M10 from a long helix starting structure at 1010 nm/s to a fully extended non-helical chain structure and then reversing the direction of pull (&#x26;ndash;108 nm/s) allowing the protein to refold (~200 ns simulation time).
