# Supplementary material for: Stable Single α-Helices Are Constant Force Springs in Proteins
Source: J Biol Chem. 2014 Aug 13;289(40):27825–35. doi: 10.1074/jbc.M114.585679 (PMC4183817; doi:10.1074/jbc.M114.585679)
Supplement: Supplemental Data [file supp_M114.585679_jbc.M114.585679-1.docx]

**Supplementary movies**

Movies are rendered so that movie frames are taken at 0.2 ns intervals during simulations. Proteins are colored from red (N-terminus) to blue (C-terminus). Zooming is applied during movies to keep the protein visible within the frame.

Movie 1: i27_no_force

Zero-force simulation of I27 from the PDB structure 1TIT (100 ns simulation time)

Movie 2: i27_stretch

Stretching I27 from a near-native starting structure at 10^8^ nm/s (120 ns simulation time)

Movie 3: SAH_no_force

Zero-force simulation of M10 SAH from a long helix starting structure (100 ns simulation time)

Movie 4: myo10_SAH

Stretching M10 from a long helix starting structure at 10^8^ nm/s (~100 ns simulation time)

Movie 5: a97_no_force

Zero-force simulation of A_97_ from a long helix starting structure (100 ns simulation time)

Movie 6: a97_stretch

Stretching A_97_ from a long helix starting structure at 10^8^ nm/s (~90 ns simulation time)

Movie 7: g97_no_force

Zero-force simulation of G_97_ from a long helix starting structure (100 ns simulation time)

Movie 8: g97_stretch

Stretching G_97_ from a collapsed coil starting structure at 10^8^ nm/s (100 ns simulation time)

Movie 9: myo10_stretch_relax

Stretching M10 from a long helix starting structure at 10^8^ nm/s and then reversing the direction of pull (–10^8^ nm/s) allowing the protein to refold (~200 ns simulation time)

Movie 10: myo10_full_stretch_relax

Stretching M10 from a long helix starting structure at 10^10^ nm/s to a fully extended non-helical chain structure and then reversing the direction of pull (–10^8^ nm/s) allowing the protein to refold (~200 ns simulation time).
